# {1,8-Bis[2-(2-oxidobenzyl­idene­amino)phen­oxy]-3,6-dioxaocta­ne}nitrato­praseodymium(III) trichloro­methane solvate

**DOI:** 10.1107/S1600536808021259

**Published:** 2008-07-31

**Authors:** Wei-Sheng Liu, Hui-Juan Wang, Xiao-Liang Tang, Zhi-Peng Zang, Da-Qi Wang

**Affiliations:** a Department of Chemistry, State Key Laboratory of Applied Organic Chemistry, College of Chemical Engineering, Lanzhou University, Lanzhou 730000, People’s Republic of China; b State Key Laboratory of Coordination Chemistry, Nanjing University, Nanjing 210093, People’s Republic of China; c Department of Chemistry, Liaocheng University, Liaocheng 252000, People’s Republic of China

## Abstract

In the title compound, [Pr(C_32_H_30_N_2_O_6_)(NO_3_)]·CHCl_3_, the Pr^III^ ion is ten-coordinated by eight O atoms and two N atoms from the acyclic crown-type Schiff base ligand and the bidentate nitrate group. The coordination polyhedron around Pr^III^ is a distorted bicapped square anti­prism. The chloro­form solvent mol­ecule is not involved either in coordination to the Pr^III^ center or in hydrogen bonding to the complex. The Pr—O(phenolate) bonds are significantly shorter than the Pr—O(ether) and Pr—O(nitrate) bonds, which suggests that the Pr—O(phenolate) bond is stronger than these other bonds. In the crystal structure, the acyclic crown-type Schiff base ligand wraps around the Pr^III^ centre, forming a pseudo-ring.

## Related literature

For general backgound, see: Wen *et al.* (2001 ▶); Liu *et al.* (2004 ▶). For related structures, see: Yu *et al.* (2006 ▶); Ding *et al.* (2007 ▶). For related literature, see: Si *et al.* (1994 ▶).
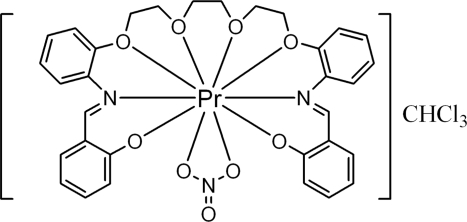


## Experimental

### 

#### Crystal data

[Pr(C_32_H_30_N_2_O_6_)(NO_3_)]·CHCl_3_
*M* *_r_* = 860.87
Monoclinic, 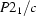

*a* = 11.3454 (14) Å
*b* = 20.150 (2) Å
*c* = 15.4676 (17) Å
β = 100.585 (2)°
*V* = 3475.9 (7) Å^3^
*Z* = 4
Mo *K*α radiation
μ = 1.69 mm^−1^
*T* = 298 (2) K
0.48 × 0.43 × 0.21 mm

#### Data collection

Bruker SMART 1000 CCD diffractometer
Absorption correction: multi-scan (*SADABS*; Sheldrick, 1996 ▶) *T*_min_ = 0.498, *T*_max_ = 0.718
17233 measured reflections
6118 independent reflections
4284 reflections with *I* > 2σ(*I*)
*R*_int_ = 0.043

#### Refinement

*R*[*F* ^2^ > 2σ(*F* ^2^)] = 0.035
*wR*(*F* ^2^) = 0.083
*S* = 1.03
6118 reflections
442 parameters
H-atom parameters constrained
Δρ_max_ = 1.44 e Å^−3^
Δρ_min_ = −0.55 e Å^−3^

### 

Data collection: *SMART* (Bruker, 1997 ▶); cell refinement: *SAINT* (Bruker, 1997 ▶); data reduction: *SAINT*; program(s) used to solve structure: *SHELXS97* (Sheldrick, 2008 ▶); program(s) used to refine structure: *SHELXL97* (Sheldrick, 2008 ▶); molecular graphics: *SHELXTL* (Sheldrick, 2008 ▶); software used to prepare material for publication: *publCIF* (Westrip, 2008 ▶).

## Supplementary Material

Crystal structure: contains datablocks I, global. DOI: 10.1107/S1600536808021259/wn2255sup1.cif

Structure factors: contains datablocks I. DOI: 10.1107/S1600536808021259/wn2255Isup2.hkl

Additional supplementary materials:  crystallographic information; 3D view; checkCIF report

## Figures and Tables

**Table:** 

Pr1—O6	2.269 (3)
Pr1—O5	2.278 (3)
Pr1—N1	2.646 (3)
Pr1—O8	2.649 (3)
Pr1—O7	2.649 (3)
Pr1—N2	2.670 (4)
Pr1—O1	2.708 (3)
Pr1—O2	2.710 (3)
Pr1—O3	2.787 (3)
Pr1—O4	2.801 (3)

**Table:** 

O6—Pr1—N1	79.26 (11)
O6—Pr1—O7	76.36 (11)
O8—Pr1—O7	47.81 (10)
O5—Pr1—N2	77.19 (11)
N1—Pr1—N2	79.07 (10)
O7—Pr1—N2	92.76 (10)
N1—Pr1—O1	59.47 (9)
O1—Pr1—O2	60.33 (8)
O2—Pr1—O3	60.76 (9)
O5—Pr1—O4	73.42 (9)
N2—Pr1—O4	56.72 (9)
O3—Pr1—O4	60.53 (8)

**Fig. 1.**
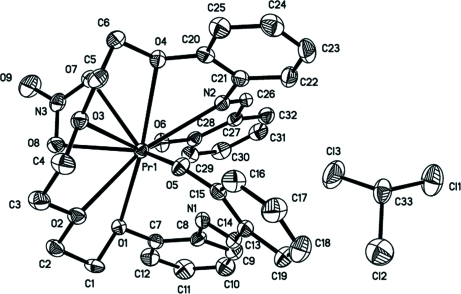
The structure of the title compound, showing the atom-labelling scheme. Displacement ellipsoids are drawn at the 30% probability level. H atoms have been omitted for clarity.

**Fig. 2.**
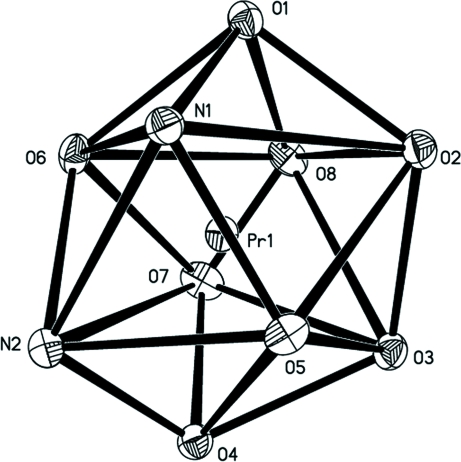
The coordination polyhedron of the title compound, showing the distorted bicapped square antiprism.

## supplementary crystallographic information

### Comment

Open chain polyethers offer many advantages over traditional crown ethers (Liu *et al.*,2004). They are excellent reagents for activating ion-selective electrodes and extracts of rare earth ions (Wen *et al.*, 2001). In recent years the structures and properties of complexes with the zinc(II) ion, rare earth ions, and non-cyclic crown-type Schiff bases have been reported (Ding *et al.*, 2007; Yu *et al.*, 2006). To further understand the ability of these compounds to complex rare earth ions, we have prepared a non-cyclic crown-type Schiff base,1,8-bis[2-(2-hydroxyphenylideneimino)phenoxy]-3,6-dioxaoctane (H_2_L), as a ligand and investigated the reaction of H_2_L with Pr(NO_3_)_3_.6H_2_O. As part of a series of studies, we report here the crystal structure of the title compound. The structure of the complex is illustrated in Fig.1. Selected bond lengths and angles are given in Table 1. The Pr^III^ ion is coordinated by ten donor atoms, eight of which belong to the non-cyclic crown-type Schiff base ligand and the remaining two to the bidentate nitrate group. The coordination polyhedron around Pr^III^ is a distorted bicapped square antiprism (Fig. 2). The chloroform solvent molecule is not involved either in coordination to the Pr^III^ center or in hydrogen bonding to the complex. The Pr—O (phenolate) bonds are stronger than the other Pr—O bonds. In the crystal structure, the non-cyclic crown-type Schiff base ligand wraps around the Pr^III^ centre, forming a pseudo-ring.

### Experimental

H_2_L was synthesized using a literature method (Si *et al.*,1994). The title compound Pr(NO_3_)(C_32_H_30_O_6_N_2_)(CHCl_3_) was synthesized as follows: NaOH (8.0 mg, 0.2 mmol) was added to 10 ml of ethyl acetate solution containing H_2_L (54.0 mg, 0.1 mmol). The mixture was stirred for 10 min at room temperature to obtain a yellow solution. 5 ml of ethyl acetate solution containing Pr(NO_3_)_3_.6H_2_O (43.4 mg, 0.1 mmol) was then added to the mixture and a yellow precipitate formed. The precipitate was collected and washed three times with ethyl acetate. Further drying in a vacuum afforded a yellow powder. Yellow single crystals of the title compound were grown from a mixed methanol/chloroform solution (v:v 1:2) by slow evaporation at room temperature.

### Refinement

All H atoms were placed in geometrically idealized positions and constrained to ride on their parent atoms, with C—H = 0.93–0.98 Å, and with *U*_iso_(H)= 1.2*U*_eq_(C). The highest residual electron density peak is located 1.32 Å from O6.

### Crystal data

**Table:**

[Pr(C_32_H_30_N_2_O_6_)(NO_3_)]·CHCl_3_	*F*_000_ = 1728
*M**_r_* = 860.87	*D*_x_ = 1.645 Mg m^−^^3^
Monoclinic, *P*2_1_/*c*	Mo *K*α radiation λ = 0.71073 Å
*a* = 11.3454 (14) Å	Cell parameters from 5219 reflections
*b* = 20.150 (2) Å	θ = 2.3–25.5º
*c* = 15.4676 (17) Å	µ = 1.69 mm^−^^1^
β = 100.585 (2)º	*T* = 298 (2) K
*V* = 3475.9 (7) Å^3^	Block, yellow
*Z* = 4	0.48 × 0.43 × 0.21 mm

### Data collection

**Table:**

Bruker SMART 1000 CCD diffractometer	6118 independent reflections
Radiation source: fine-focus sealed tube	4284 reflections with *I* > 2σ(*I*)
Monochromator: graphite	*R*_int_ = 0.043
*T* = 298(2) K	θ_max_ = 25.0º
φ and ω scans	θ_min_ = 1.7º
Absorption correction: multi-scan(SADABS; Sheldrick, 1996)	*h* = −13→13
*T*_min_ = 0.498, *T*_max_ = 0.718	*k* = −23→20
17233 measured reflections	*l* = −16→18

### Refinement

**Table:**

Refinement on *F*^2^	Secondary atom site location: difference Fourier map
Least-squares matrix: full	Hydrogen site location: inferred from neighbouring sites
*R*[*F*^2^ > 2σ(*F*^2^)] = 0.035	H-atom parameters constrained
*wR*(*F*^2^) = 0.083	*w* = 1/[σ^2^(*F*_o_^2^) + (0.0368*P*)^2^] where *P* = (*F*_o_^2^ + 2*F*_c_^2^)/3
*S* = 1.03	(Δ/σ)_max_ = 0.001
6118 reflections	Δρ_max_ = 1.44 e Å^−^^3^
442 parameters	Δρ_min_ = −0.55 e Å^−^^3^
Primary atom site location: structure-invariant direct methods	Extinction correction: none

### Special details

**Table:**

Geometry. All e.s.d.'s (except the e.s.d. in the dihedral angle between two l.s. planes) are estimated using the full covariance matrix. The cell e.s.d.'s are taken into account individually in the estimation of e.s.d.'s in distances, angles and torsion angles; correlations between e.s.d.'s in cell parameters are only used when they are defined by crystal symmetry. An approximate (isotropic) treatment of cell e.s.d.'s is used for estimating e.s.d.'s involving l.s. planes.
Refinement. Refinement of *F*^2^ against ALL reflections. The weighted *R*-factor *wR* and goodness of fit *S* are based on *F*^2^, conventional *R*-factors *R* are based on *F*, with *F* set to zero for negative *F*^2^. The threshold expression of *F*^2^ > σ(*F*^2^) is used only for calculating *R*-factors(gt) *etc*. and is not relevant to the choice of reflections for refinement. *R*-factors based on *F*^2^ are statistically about twice as large as those based on *F*, and *R*- factors based on ALL data will be even larger.

### Fractional atomic coordinates and isotropic or equivalent isotropic displacement parameters (Å^2^)

**Table:**

	*x*	*y*	*z*	*U*_iso_*/*U*_eq_	
Pr1	0.84007 (2)	0.784583 (11)	0.158214 (16)	0.03001 (9)	
Cl1	0.16296 (15)	0.61952 (9)	0.19132 (14)	0.0999 (6)	
Cl2	0.23419 (17)	0.65708 (9)	0.03000 (13)	0.0984 (6)	
Cl3	0.41044 (14)	0.64221 (10)	0.18791 (15)	0.1164 (7)	
N1	0.6762 (3)	0.71096 (16)	0.0580 (2)	0.0339 (8)	
N2	0.6852 (3)	0.76463 (17)	0.2664 (2)	0.0348 (9)	
N3	1.1034 (3)	0.79143 (19)	0.2513 (3)	0.0436 (10)	
C9	0.6446 (4)	0.5878 (2)	0.0482 (3)	0.0503 (13)	
H9	0.5646	0.5918	0.0534	0.060*	
O1	0.9051 (2)	0.69333 (14)	0.04725 (19)	0.0372 (7)	
O2	0.9149 (3)	0.82509 (15)	0.01016 (19)	0.0409 (8)	
O3	0.9091 (3)	0.91629 (14)	0.1443 (2)	0.0389 (7)	
O4	0.8019 (2)	0.87638 (13)	0.2862 (2)	0.0376 (7)	
O5	0.6794 (2)	0.84723 (13)	0.09646 (19)	0.0381 (7)	
O6	0.8714 (2)	0.68515 (14)	0.2275 (2)	0.0394 (7)	
O7	1.0201 (3)	0.80827 (17)	0.2899 (2)	0.0514 (9)	
O8	1.0758 (3)	0.78130 (15)	0.1693 (2)	0.0462 (8)	
O9	1.2050 (3)	0.7844 (2)	0.2900 (3)	0.0823 (13)	
C1	0.9179 (4)	0.7150 (2)	−0.0398 (3)	0.0458 (12)	
H1A	0.8394	0.7200	−0.0767	0.055*	
H1B	0.9623	0.6821	−0.0666	0.055*	
C2	0.9823 (4)	0.7793 (2)	−0.0330 (3)	0.0453 (12)	
H2A	1.0627	0.7741	0.0008	0.054*	
H2B	0.9882	0.7956	−0.0911	0.054*	
C3	0.9633 (5)	0.8900 (2)	0.0084 (3)	0.0529 (14)	
H3A	0.9595	0.9042	−0.0520	0.064*	
H3B	1.0467	0.8902	0.0375	0.064*	
C4	0.8932 (5)	0.9359 (2)	0.0539 (3)	0.0541 (14)	
H4A	0.9210	0.9811	0.0495	0.065*	
H4B	0.8090	0.9339	0.0270	0.065*	
C5	0.8566 (4)	0.9631 (2)	0.1947 (3)	0.0454 (12)	
H5A	0.7723	0.9690	0.1700	0.055*	
H5B	0.8964	1.0057	0.1950	0.055*	
C6	0.8715 (4)	0.9359 (2)	0.2860 (3)	0.0438 (12)	
H6A	0.9554	0.9263	0.3080	0.053*	
H6B	0.8454	0.9686	0.3245	0.053*	
C7	0.8358 (4)	0.6361 (2)	0.0440 (3)	0.0375 (11)	
C8	0.7160 (4)	0.6445 (2)	0.0490 (3)	0.0371 (11)	
C10	0.6931 (5)	0.5260 (2)	0.0397 (4)	0.0599 (15)	
H10	0.6453	0.4884	0.0384	0.072*	
C11	0.8117 (5)	0.5196 (2)	0.0331 (4)	0.0609 (15)	
H11	0.8438	0.4778	0.0270	0.073*	
C12	0.8823 (5)	0.5746 (2)	0.0354 (3)	0.0495 (13)	
H12	0.9626	0.5701	0.0311	0.059*	
C13	0.5709 (4)	0.7263 (2)	0.0152 (3)	0.0386 (11)	
H13	0.5275	0.6923	−0.0164	0.046*	
C14	0.5133 (4)	0.7900 (2)	0.0109 (3)	0.0358 (10)	
C15	0.5697 (4)	0.8475 (2)	0.0520 (3)	0.0351 (10)	
C16	0.5031 (4)	0.9069 (2)	0.0427 (3)	0.0523 (13)	
H16	0.5364	0.9450	0.0712	0.063*	
C17	0.3912 (5)	0.9097 (3)	−0.0070 (4)	0.0635 (16)	
H17	0.3502	0.9499	−0.0129	0.076*	
C18	0.3374 (5)	0.8544 (3)	−0.0488 (4)	0.0695 (17)	
H18	0.2606	0.8568	−0.0824	0.083*	
C19	0.3990 (4)	0.7954 (3)	−0.0402 (3)	0.0525 (14)	
H19	0.3635	0.7580	−0.0692	0.063*	
C20	0.6820 (4)	0.8820 (2)	0.2903 (3)	0.0364 (11)	
C21	0.6188 (4)	0.8227 (2)	0.2810 (3)	0.0376 (11)	
C22	0.4985 (4)	0.8233 (3)	0.2833 (3)	0.0486 (13)	
H22	0.4554	0.7838	0.2758	0.058*	
C23	0.4404 (4)	0.8817 (3)	0.2966 (4)	0.0577 (15)	
H23	0.3590	0.8817	0.2987	0.069*	
C24	0.5048 (5)	0.9396 (3)	0.3066 (4)	0.0576 (15)	
H24	0.4665	0.9790	0.3163	0.069*	
C25	0.6248 (5)	0.9408 (2)	0.3027 (3)	0.0507 (13)	
H25	0.6670	0.9806	0.3082	0.061*	
C26	0.6611 (4)	0.7105 (2)	0.3046 (3)	0.0372 (11)	
H26	0.6015	0.7130	0.3385	0.045*	
C27	0.7169 (4)	0.6467 (2)	0.3000 (3)	0.0357 (11)	
C28	0.8194 (4)	0.6370 (2)	0.2618 (3)	0.0348 (11)	
C29	0.8640 (4)	0.5720 (2)	0.2616 (3)	0.0462 (12)	
H29	0.9306	0.5641	0.2358	0.055*	
C30	0.8129 (5)	0.5196 (2)	0.2980 (3)	0.0554 (14)	
H30	0.8447	0.4772	0.2963	0.066*	
C31	0.7142 (5)	0.5299 (3)	0.3372 (3)	0.0550 (14)	
H31	0.6801	0.4947	0.3625	0.066*	
C32	0.6671 (4)	0.5927 (2)	0.3383 (3)	0.0487 (13)	
H32	0.6009	0.5996	0.3649	0.058*	
C33	0.2640 (4)	0.6656 (3)	0.1447 (4)	0.0643 (16)	
H33	0.2544	0.7125	0.1589	0.077*	

### Atomic displacement parameters (Å^2^)

**Table:**

	*U*^11^	*U*^22^	*U*^33^	*U*^12^	*U*^13^	*U*^23^
Pr1	0.02794 (14)	0.03197 (14)	0.02875 (14)	−0.00197 (11)	0.00155 (9)	0.00156 (12)
Cl1	0.0752 (12)	0.0960 (13)	0.1290 (17)	−0.0180 (10)	0.0202 (11)	0.0328 (12)
Cl2	0.1104 (14)	0.0852 (12)	0.1010 (15)	−0.0041 (10)	0.0232 (11)	−0.0036 (11)
Cl3	0.0498 (10)	0.1420 (17)	0.150 (2)	0.0073 (10)	−0.0020 (11)	−0.0466 (15)
N1	0.030 (2)	0.033 (2)	0.040 (2)	−0.0011 (17)	0.0089 (17)	−0.0020 (18)
N2	0.033 (2)	0.039 (2)	0.031 (2)	−0.0044 (17)	0.0038 (16)	0.0001 (17)
N3	0.026 (2)	0.053 (3)	0.048 (3)	0.0021 (19)	−0.0038 (19)	0.002 (2)
C9	0.050 (3)	0.048 (3)	0.052 (3)	−0.010 (3)	0.009 (3)	−0.010 (3)
O1	0.0364 (18)	0.0414 (18)	0.0343 (18)	−0.0057 (14)	0.0081 (14)	0.0002 (14)
O2	0.050 (2)	0.0399 (19)	0.0327 (19)	−0.0042 (15)	0.0083 (15)	0.0018 (15)
O3	0.0424 (19)	0.0337 (17)	0.041 (2)	−0.0066 (14)	0.0079 (15)	0.0002 (15)
O4	0.0342 (18)	0.0356 (18)	0.044 (2)	−0.0018 (14)	0.0087 (14)	0.0000 (15)
O5	0.0312 (17)	0.0356 (17)	0.0435 (19)	0.0013 (14)	−0.0037 (14)	−0.0021 (15)
O6	0.0354 (18)	0.0364 (17)	0.047 (2)	0.0035 (14)	0.0083 (15)	0.0129 (16)
O7	0.039 (2)	0.069 (2)	0.045 (2)	0.0021 (17)	0.0033 (16)	−0.0087 (18)
O8	0.0381 (18)	0.065 (2)	0.0342 (19)	0.0018 (16)	0.0036 (15)	−0.0025 (17)
O9	0.037 (2)	0.128 (4)	0.072 (3)	0.006 (2)	−0.015 (2)	−0.009 (3)
C1	0.061 (3)	0.052 (3)	0.025 (3)	0.000 (3)	0.010 (2)	−0.005 (2)
C2	0.060 (3)	0.043 (3)	0.036 (3)	−0.006 (2)	0.018 (2)	0.004 (2)
C3	0.079 (4)	0.042 (3)	0.040 (3)	−0.012 (3)	0.017 (3)	0.004 (2)
C4	0.087 (4)	0.032 (3)	0.046 (3)	−0.006 (3)	0.021 (3)	0.009 (2)
C5	0.053 (3)	0.028 (3)	0.054 (3)	−0.010 (2)	0.007 (3)	−0.007 (2)
C6	0.048 (3)	0.038 (3)	0.046 (3)	−0.008 (2)	0.009 (2)	−0.007 (2)
C7	0.043 (3)	0.036 (3)	0.035 (3)	−0.007 (2)	0.010 (2)	−0.002 (2)
C8	0.037 (3)	0.035 (3)	0.038 (3)	−0.004 (2)	0.002 (2)	−0.008 (2)
C10	0.077 (4)	0.039 (3)	0.066 (4)	−0.018 (3)	0.020 (3)	−0.010 (3)
C11	0.078 (4)	0.031 (3)	0.076 (4)	0.005 (3)	0.018 (3)	−0.008 (3)
C12	0.055 (3)	0.036 (3)	0.058 (4)	0.008 (2)	0.015 (3)	−0.008 (3)
C13	0.035 (3)	0.041 (3)	0.039 (3)	−0.011 (2)	0.005 (2)	−0.007 (2)
C14	0.031 (2)	0.043 (3)	0.033 (3)	−0.001 (2)	0.005 (2)	0.002 (2)
C15	0.032 (3)	0.045 (3)	0.030 (3)	0.003 (2)	0.008 (2)	0.003 (2)
C16	0.055 (3)	0.049 (3)	0.051 (3)	0.012 (3)	0.004 (3)	0.002 (3)
C17	0.049 (3)	0.071 (4)	0.065 (4)	0.026 (3)	−0.005 (3)	−0.003 (3)
C18	0.034 (3)	0.093 (5)	0.074 (4)	0.017 (3)	−0.008 (3)	0.004 (4)
C19	0.037 (3)	0.066 (4)	0.050 (3)	−0.003 (3)	−0.004 (2)	−0.002 (3)
C20	0.039 (3)	0.039 (3)	0.031 (3)	0.007 (2)	0.007 (2)	0.001 (2)
C21	0.038 (3)	0.041 (3)	0.033 (3)	0.003 (2)	0.006 (2)	−0.002 (2)
C22	0.035 (3)	0.054 (3)	0.056 (3)	−0.003 (2)	0.007 (2)	−0.002 (3)
C23	0.036 (3)	0.072 (4)	0.068 (4)	0.007 (3)	0.016 (3)	0.004 (3)
C24	0.056 (4)	0.056 (4)	0.062 (4)	0.019 (3)	0.014 (3)	−0.003 (3)
C25	0.055 (3)	0.046 (3)	0.052 (3)	0.000 (3)	0.011 (3)	0.000 (3)
C26	0.029 (2)	0.054 (3)	0.030 (2)	−0.010 (2)	0.0087 (19)	−0.009 (2)
C27	0.041 (3)	0.033 (3)	0.031 (3)	−0.008 (2)	0.002 (2)	−0.001 (2)
C28	0.033 (3)	0.036 (3)	0.032 (3)	−0.003 (2)	−0.002 (2)	0.005 (2)
C29	0.050 (3)	0.046 (3)	0.043 (3)	0.006 (2)	0.009 (2)	0.005 (2)
C30	0.068 (4)	0.042 (3)	0.052 (4)	0.000 (3)	−0.001 (3)	0.005 (3)
C31	0.073 (4)	0.044 (3)	0.045 (3)	−0.020 (3)	0.004 (3)	0.008 (3)
C32	0.050 (3)	0.051 (3)	0.044 (3)	−0.013 (3)	0.007 (2)	0.003 (3)
C33	0.046 (3)	0.048 (3)	0.100 (5)	−0.001 (3)	0.016 (3)	−0.010 (3)

### Geometric parameters (Å, °)

**Table:**

Pr1—O6	2.269 (3)	C5—H5B	0.9700
Pr1—O5	2.278 (3)	C6—H6A	0.9700
Pr1—N1	2.646 (3)	C6—H6B	0.9700
Pr1—O8	2.649 (3)	C7—C12	1.363 (6)
Pr1—O7	2.649 (3)	C7—C8	1.386 (6)
Pr1—N2	2.670 (4)	C10—C11	1.374 (7)
Pr1—O1	2.708 (3)	C10—H10	0.9300
Pr1—O2	2.710 (3)	C11—C12	1.364 (6)
Pr1—O3	2.787 (3)	C11—H11	0.9300
Pr1—O4	2.801 (3)	C12—H12	0.9300
Cl1—C33	1.733 (5)	C13—C14	1.436 (6)
Cl2—C33	1.752 (6)	C13—H13	0.9300
Cl3—C33	1.738 (5)	C14—C19	1.393 (6)
N1—C13	1.293 (5)	C14—C15	1.415 (6)
N1—C8	1.429 (5)	C15—C16	1.408 (6)
N2—C26	1.293 (5)	C16—C17	1.360 (7)
N2—C21	1.432 (5)	C16—H16	0.9300
N3—O9	1.206 (5)	C17—C18	1.374 (7)
N3—O7	1.255 (4)	C17—H17	0.9300
N3—O8	1.266 (5)	C18—C19	1.372 (7)
C9—C10	1.378 (6)	C18—H18	0.9300
C9—C8	1.399 (6)	C19—H19	0.9300
C9—H9	0.9300	C20—C25	1.379 (6)
O1—C7	1.391 (5)	C20—C21	1.388 (6)
O1—C1	1.448 (5)	C21—C22	1.372 (6)
O2—C3	1.421 (5)	C22—C23	1.382 (6)
O2—C2	1.438 (5)	C22—H22	0.9300
O3—C5	1.422 (5)	C23—C24	1.371 (7)
O3—C4	1.432 (5)	C23—H23	0.9300
O4—C20	1.378 (5)	C24—C25	1.374 (6)
O4—C6	1.437 (5)	C24—H24	0.9300
O5—C15	1.306 (5)	C25—H25	0.9300
O6—C28	1.298 (5)	C26—C27	1.440 (6)
C1—C2	1.482 (6)	C26—H26	0.9300
C1—H1A	0.9700	C27—C32	1.407 (6)
C1—H1B	0.9700	C27—C28	1.411 (6)
C2—H2A	0.9700	C28—C29	1.405 (6)
C2—H2B	0.9700	C29—C30	1.373 (6)
C3—C4	1.480 (6)	C29—H29	0.9300
C3—H3A	0.9700	C30—C31	1.383 (7)
C3—H3B	0.9700	C30—H30	0.9300
C4—H4A	0.9700	C31—C32	1.375 (7)
C4—H4B	0.9700	C31—H31	0.9300
C5—C6	1.495 (6)	C32—H32	0.9300
C5—H5A	0.9700	C33—H33	0.9800
			
O6—Pr1—O5	136.97 (10)	C3—C4—H4B	110.1
O6—Pr1—N1	79.26 (11)	H4A—C4—H4B	108.4
O5—Pr1—N1	69.09 (10)	O3—C5—C6	106.8 (4)
O6—Pr1—O8	83.05 (10)	O3—C5—H5A	110.4
O5—Pr1—O8	139.60 (10)	C6—C5—H5A	110.4
N1—Pr1—O8	128.04 (10)	O3—C5—H5B	110.4
O6—Pr1—O7	76.36 (11)	C6—C5—H5B	110.4
O5—Pr1—O7	131.84 (10)	H5A—C5—H5B	108.6
N1—Pr1—O7	155.61 (11)	O4—C6—C5	109.9 (4)
O8—Pr1—O7	47.81 (10)	O4—C6—H6A	109.7
O6—Pr1—N2	68.58 (10)	C5—C6—H6A	109.7
O5—Pr1—N2	77.19 (11)	O4—C6—H6B	109.7
N1—Pr1—N2	79.07 (10)	C5—C6—H6B	109.7
O8—Pr1—N2	136.75 (10)	H6A—C6—H6B	108.2
O7—Pr1—N2	92.76 (10)	C12—C7—C8	121.2 (4)
O6—Pr1—O1	70.29 (10)	C12—C7—O1	122.1 (4)
O5—Pr1—O1	113.61 (10)	C8—C7—O1	116.8 (4)
N1—Pr1—O1	59.47 (9)	C7—C8—C9	118.2 (4)
O8—Pr1—O1	68.58 (9)	C7—C8—N1	116.8 (4)
O7—Pr1—O1	110.38 (9)	C9—C8—N1	125.0 (4)
N2—Pr1—O1	125.60 (9)	C11—C10—C9	120.3 (5)
O6—Pr1—O2	128.24 (10)	C11—C10—H10	119.8
O5—Pr1—O2	80.19 (10)	C9—C10—H10	119.8
N1—Pr1—O2	88.25 (10)	C12—C11—C10	120.1 (5)
O8—Pr1—O2	65.98 (9)	C12—C11—H11	119.9
O7—Pr1—O2	106.17 (10)	C10—C11—H11	119.9
N2—Pr1—O2	156.82 (10)	C7—C12—C11	120.3 (5)
O1—Pr1—O2	60.33 (8)	C7—C12—H12	119.9
O6—Pr1—O3	148.83 (10)	C11—C12—H12	119.9
O5—Pr1—O3	69.79 (9)	N1—C13—C14	127.4 (4)
N1—Pr1—O3	131.76 (10)	N1—C13—H13	116.3
O8—Pr1—O3	74.57 (9)	C14—C13—H13	116.3
O7—Pr1—O3	72.54 (10)	C19—C14—C15	119.2 (4)
N2—Pr1—O3	114.75 (10)	C19—C14—C13	117.6 (4)
O1—Pr1—O3	118.97 (8)	C15—C14—C13	123.1 (4)
O2—Pr1—O3	60.76 (9)	O5—C15—C16	119.9 (4)
O6—Pr1—O4	106.28 (10)	O5—C15—C14	122.8 (4)
O5—Pr1—O4	73.42 (9)	C16—C15—C14	117.4 (4)
N1—Pr1—O4	126.95 (9)	C17—C16—C15	121.4 (5)
O8—Pr1—O4	104.85 (9)	C17—C16—H16	119.3
O7—Pr1—O4	62.29 (9)	C15—C16—H16	119.3
N2—Pr1—O4	56.72 (9)	C16—C17—C18	121.4 (5)
O1—Pr1—O4	172.66 (8)	C16—C17—H17	119.3
O2—Pr1—O4	120.76 (8)	C18—C17—H17	119.3
O3—Pr1—O4	60.53 (8)	C19—C18—C17	118.8 (5)
C13—N1—C8	117.0 (4)	C19—C18—H18	120.6
C13—N1—Pr1	130.5 (3)	C17—C18—H18	120.6
C8—N1—Pr1	112.5 (2)	C18—C19—C14	121.8 (5)
C26—N2—C21	117.1 (4)	C18—C19—H19	119.1
C26—N2—Pr1	129.2 (3)	C14—C19—H19	119.1
C21—N2—Pr1	113.7 (3)	O4—C20—C25	124.8 (4)
O9—N3—O7	122.1 (4)	O4—C20—C21	114.8 (4)
O9—N3—O8	121.1 (4)	C25—C20—C21	120.4 (4)
O7—N3—O8	116.8 (4)	C22—C21—C20	119.1 (4)
C10—C9—C8	119.9 (5)	C22—C21—N2	124.5 (4)
C10—C9—H9	120.1	C20—C21—N2	116.3 (4)
C8—C9—H9	120.1	C21—C22—C23	121.0 (5)
C7—O1—C1	111.6 (3)	C21—C22—H22	119.5
C7—O1—Pr1	111.6 (2)	C23—C22—H22	119.5
C1—O1—Pr1	118.0 (2)	C24—C23—C22	118.9 (5)
C3—O2—C2	109.9 (3)	C24—C23—H23	120.6
C3—O2—Pr1	118.4 (3)	C22—C23—H23	120.6
C2—O2—Pr1	118.3 (2)	C23—C24—C25	121.4 (5)
C5—O3—C4	111.2 (3)	C23—C24—H24	119.3
C5—O3—Pr1	115.9 (2)	C25—C24—H24	119.3
C4—O3—Pr1	110.6 (2)	C24—C25—C20	119.1 (5)
C20—O4—C6	118.6 (3)	C24—C25—H25	120.4
C20—O4—Pr1	111.7 (2)	C20—C25—H25	120.4
C6—O4—Pr1	113.2 (2)	N2—C26—C27	126.7 (4)
C15—O5—Pr1	146.5 (3)	N2—C26—H26	116.6
C28—O6—Pr1	144.1 (3)	C27—C26—H26	116.6
N3—O7—Pr1	97.3 (3)	C32—C27—C28	119.7 (4)
N3—O8—Pr1	97.0 (2)	C32—C27—C26	117.1 (4)
O1—C1—C2	109.2 (4)	C28—C27—C26	123.2 (4)
O1—C1—H1A	109.8	O6—C28—C29	120.4 (4)
C2—C1—H1A	109.8	O6—C28—C27	122.6 (4)
O1—C1—H1B	109.8	C29—C28—C27	117.1 (4)
C2—C1—H1B	109.8	C30—C29—C28	122.4 (5)
H1A—C1—H1B	108.3	C30—C29—H29	118.8
O2—C2—C1	107.2 (4)	C28—C29—H29	118.8
O2—C2—H2A	110.3	C29—C30—C31	120.1 (5)
C1—C2—H2A	110.3	C29—C30—H30	120.0
O2—C2—H2B	110.3	C31—C30—H30	120.0
C1—C2—H2B	110.3	C32—C31—C30	119.4 (5)
H2A—C2—H2B	108.5	C32—C31—H31	120.3
O2—C3—C4	108.8 (4)	C30—C31—H31	120.3
O2—C3—H3A	109.9	C31—C32—C27	121.3 (5)
C4—C3—H3A	109.9	C31—C32—H32	119.4
O2—C3—H3B	109.9	C27—C32—H32	119.4
C4—C3—H3B	109.9	Cl1—C33—Cl3	110.7 (3)
H3A—C3—H3B	108.3	Cl1—C33—Cl2	110.4 (3)
O3—C4—C3	108.0 (4)	Cl3—C33—Cl2	110.9 (3)
O3—C4—H4A	110.1	Cl1—C33—H33	108.2
C3—C4—H4A	110.1	Cl3—C33—H33	108.2
O3—C4—H4B	110.1	Cl2—C33—H33	108.2
